# Four-week individual caging of male ICR mice alters body composition without change in body mass

**DOI:** 10.1038/s41598-018-19808-x

**Published:** 2018-01-22

**Authors:** Jisu Shin, Jiwan Woo, Yakdol Cho, Yoon Hee Choi, Naewoo Neo Shin, YoungSoo Kim

**Affiliations:** 1 Department of Pharmacy, Yonsei University, 85 Songdogwahak-ro, Yeonsu-gu, Incheon, 21983 South Korea; 2 Research Animal Resource Center, Korea Institute of Science and Technology (KIST), Hwarang-ro 14-gil 5, Seongbuk-gu, Seoul, 136-791 South Korea; 3 Integrated Science and Engineering Division, Yonsei University, 85 Songdogwahak-ro, Yeonsu-gu, Veritas Hall D411, Incheon, 21983 South Korea

## Abstract

Understanding the physiological implications of caging conditions for mice is crucial in improving the replicability and reliability of animal research. Individual caging of mice is known to alter mouse psychology, such as triggering depression-like symptoms in mice, suggesting that caging conditions could have negative effects on mice. Therefore, we hypothesized that individual caging could affect the physical composition of outbred mice. To investigate this, dual X-ray absorptiometry (DXA) was used to compare the mass, bone mineral content (BMC), bone mineral density (BMD), lean tissue percentage and fat tissue percentage between group and individual caged mice. We also conducted open field test to compare mouse activities in different caging conditions. Our results showed significantly reduced BMD and lean tissue percentage and significantly increased fat tissue percentage in individually-caged male mice. Furthermore, there were no differences in body mass and activity between the grouped and individual mice, suggesting that these physical alterations were not induced by group-related activity. In this study, we conclude that individual caging could alter the body composition of mice without affecting external morphology.

## Introduction

For drug discovery research, most drug candidates have been administered in animals using a mg/kg approach, where the dosage of the drug is determined by the animal’s body weight. The efficiency of drugs is dependent on four fundamental pathways characterized as the ADME process – absorption into the bloodstream, distribution to the tissues, metabolism of drug particles, and excretion from the body^1^. One of the main factors that contribute to ADME is body composition^2,3^; therefore, changes in the body composition can lead to unreliable results of animal testing.

Mice, being social animals, are thought to be negatively affected by isolation^4^. This has been supported by a previous study that has shown depression-like behavior in individually-caged mice^5^. While the psychological implications of solitude in mice have been relatively well established, the implications of social isolation on the body composition parameters of mice remain uncharacterized.

Therefore, to investigate the effect of group or individually caging on the physical composition of mice, we conducted a study comparing the body composition parameters of all mice. In this study, all eight-week-old ICR male mice (*n* = 22) were caged in groups (*n* = 12, 4 per cage) or individually (*n* = 10, 1 per cage) for four weeks. This study provides evidence of a significant differences in bodily content in group-caged and individually-caged mice, highlighting the influence of such external factors on mice physiology. We obtained the values of body mass, BMC, BMD, lean and fat tissue from InAlyzer using DXA analysis (Medikors Inc., Korea). Then we measured the locomotive activity of mice (*n* = 20) after four weeks of caging in group- (*n* = 16, 4 per cage) or individually-caged (*n* = 4, 1 per cage).

## Results

### BMD and lean percentage is decreased, fat percentage is increased in individually-caged mice

**Figure 1:**
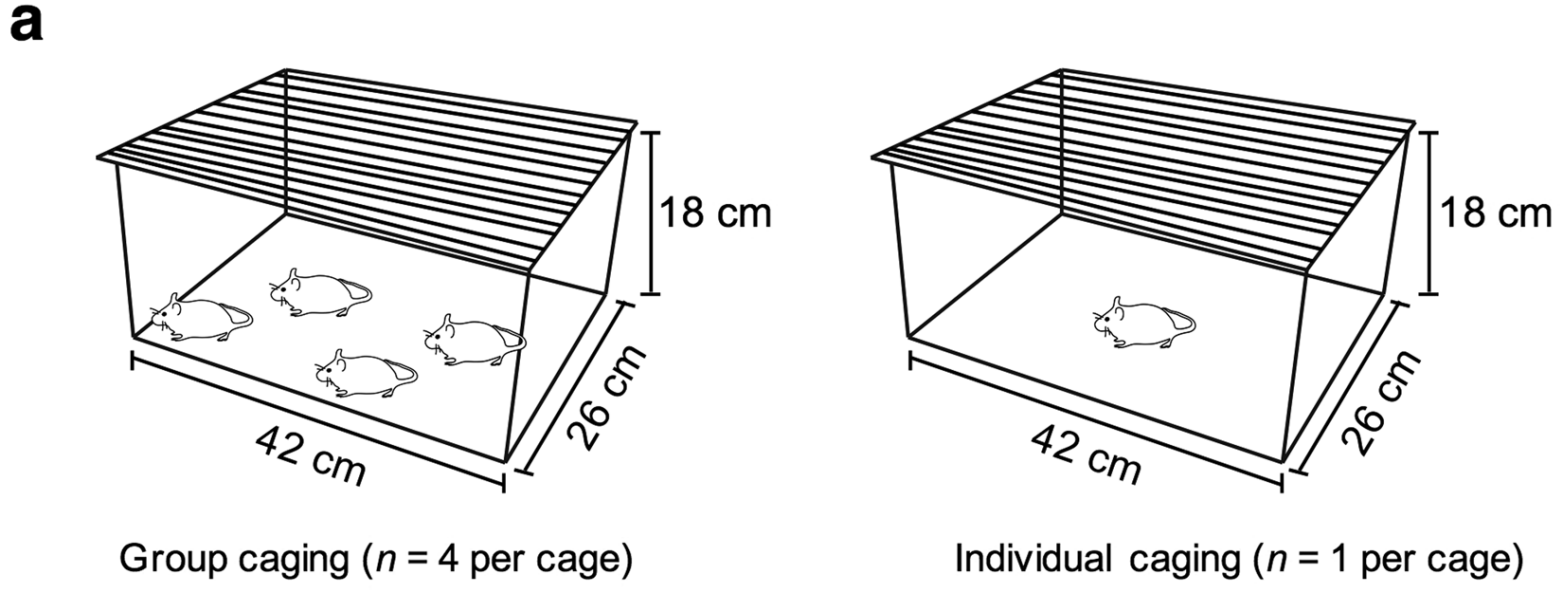
All mice were caged individually or together for four weeks. Eight-week-old male ICR mice (*n* = 22) were caged in groups of four or individually (*n* = 12 and *n* = 10, respectively).

**Figure 2:**
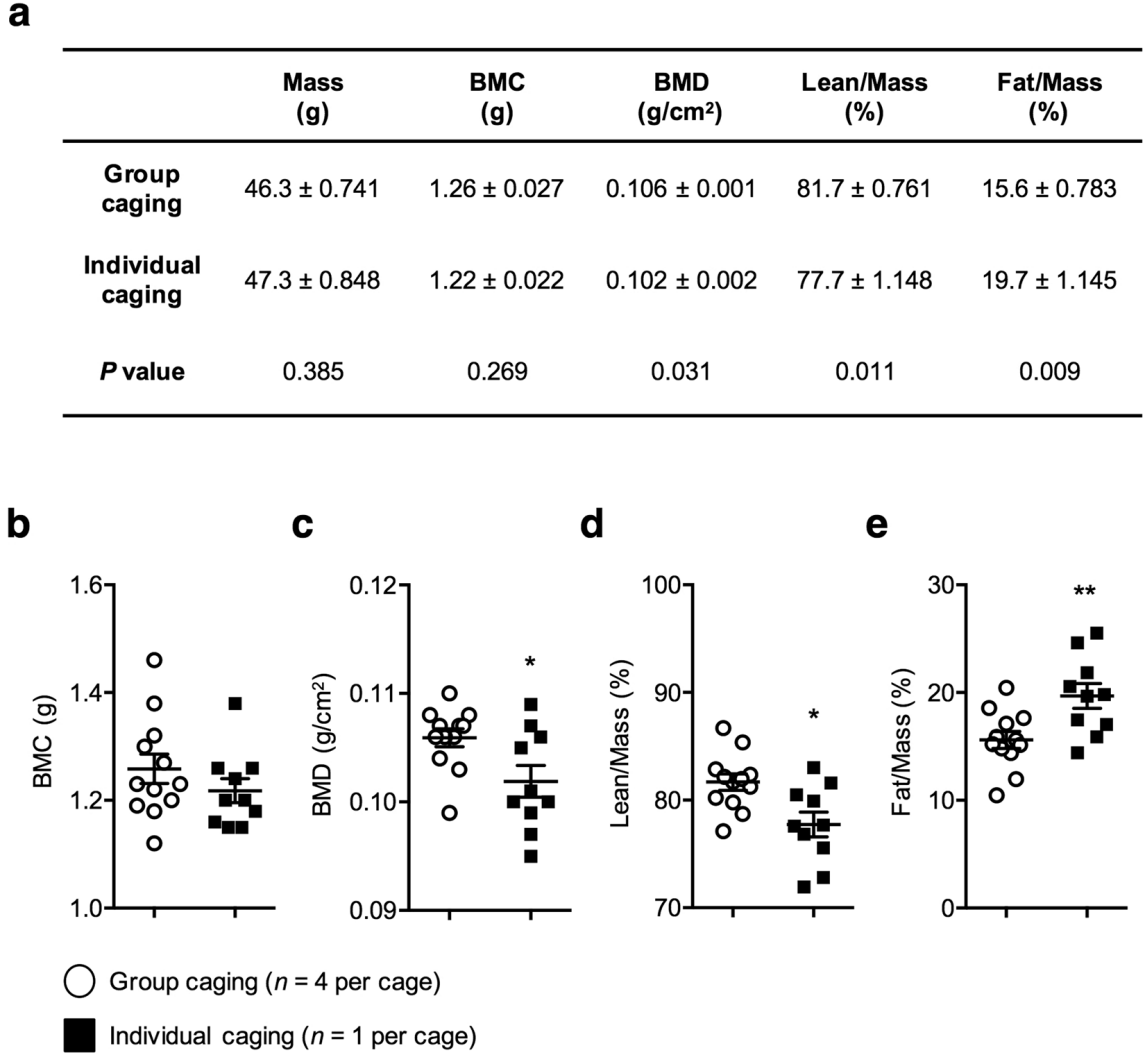
Body composition differences between group-caged and individually-caged mice. After four weeks of caging, bone mineral content (BMC), bone mineral density (BMD), lean tissue percentage and fat tissue percentage of mice were measured using InAlyzer. **(a)** Table of the mean BMC and BMD, and mean percentage mass of the parameters lean tissue and fat tissue relative to body mass. **(b)** BMC value (**g**). BMC of both groups of mice were not significant (*t*-test*, P* = 0.269). **(c)** BMD value (g/cm^2^). Compared to the group-caged mice, individual caged mice have significantly lower BMD (*t*-test*, P* = 0.0031). **(d)** Lean tissue percentage to mass (%). Compared to the group-caged mice, individual caged mice have significantly lower lean tissue percentage (*t*-test*, P* = 0.011). **(e)** Fat tissue percentage to mass (%). Compared to the group-caged mice, individually-caged mice have significantly higher fat percentage (*t*-test*, P* = 0.009). All the error bars represent the SEM. (*P < 0.05, **P < 0.01, ***P < 0.001).

After four weeks of caging in groups or individually (Fig. 1a), the physical effects of caging were observed. To assess these effects, the measurements of body mass, BMD, BMC, lean tissue percentage, and fat tissue percentage were collected and compared. Absolute values and the means of each measurement criteria were obtained using the InAlyzer (Supplementary Tables S1, S2 and S3). Because the mice varied in mass, we measured the proportion of the lean tissue and fat tissue mass to the total body mass and calculated the mean of each percentage value for accurate comparison (Fig. 2a). There was no significant difference in body mass and BMC (*t*-test*, P* = 0.269, Fig. 2b) between the individually-caged and group-caged mice, however, the test parameters of BMD, lean tissue percentage, and fat tissue percentage were significantly different between group-caged (*n* = 4 per cage) and individually-caged (*n* = 1 per cage) mice. Compared to grouped mice, individual mice showed a significantly lower BMD value (*t*-test*, P* = 0.031, Fig. 2c) as well as a lower percentage for lean tissue (*t*-test*, P* = 0.011, Fig. 2d). Furthermore, fat tissue percentage was shown to significantly increase in mice caged individually (*t*-test, *P* = 0.009, Fig. 2e).

### Increase in fat distribution is observed in individually-caged mice

**Figure 3:**
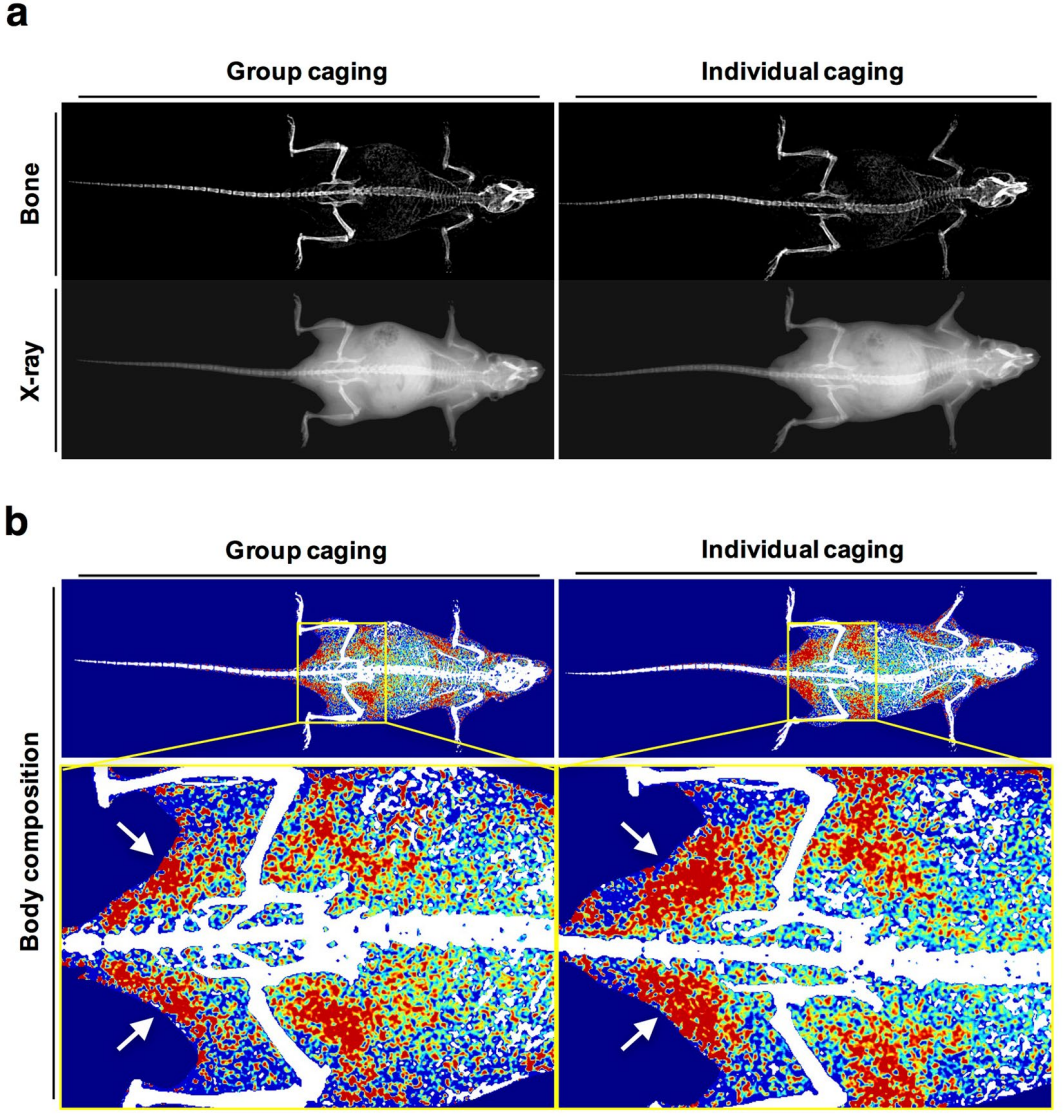
Representative images of bone, X-ray and body composition of group-caged and individually-caged mice. Images obtained from the InAlyzer during measurement. **(a)** Bone and X-ray images of mice. No observable differences between groups and abnormalities are seen in either group. **(b)** Representative body composition images of group-caged and individually-caged mice (red = fat tissue, blue = lean tissue). In the enlarged image (yellow box), a greater fat composition, indicated in red, is observed in individually-caged mice (white arrows).

We obtained the images of bone, X-ray, and body composition from DXA analysis and further analyzed the physical effects of individual caging on mice. From the bone and X-ray images, no physical abnormalities or changes in morphology could be observed in both the group-caged (*n* = 12, 4 per cage) and individually-caged mice (*n* = 10, 1 per cage) and no prominent difference in bone formation or tissue distribution could be observed from the scanned images (Fig. 3a, Supplementary Figures S1 and S2). There are no changes in hair loss of mice, which is a common indicator of poor health, in both individual and group caging after four weeks of caging. However, a markedly low distribution of fat, indicated with red in the body composition image, in individually-caged mice compared to group-caged mice was observed (Fig. 3b, indicated by white arrows). The differences in the body composition images suggest a physical effect on mice due to isolation.

### Alterations in body composition parameters of individually-caged mice is not affected by activity

**Figure 4:**
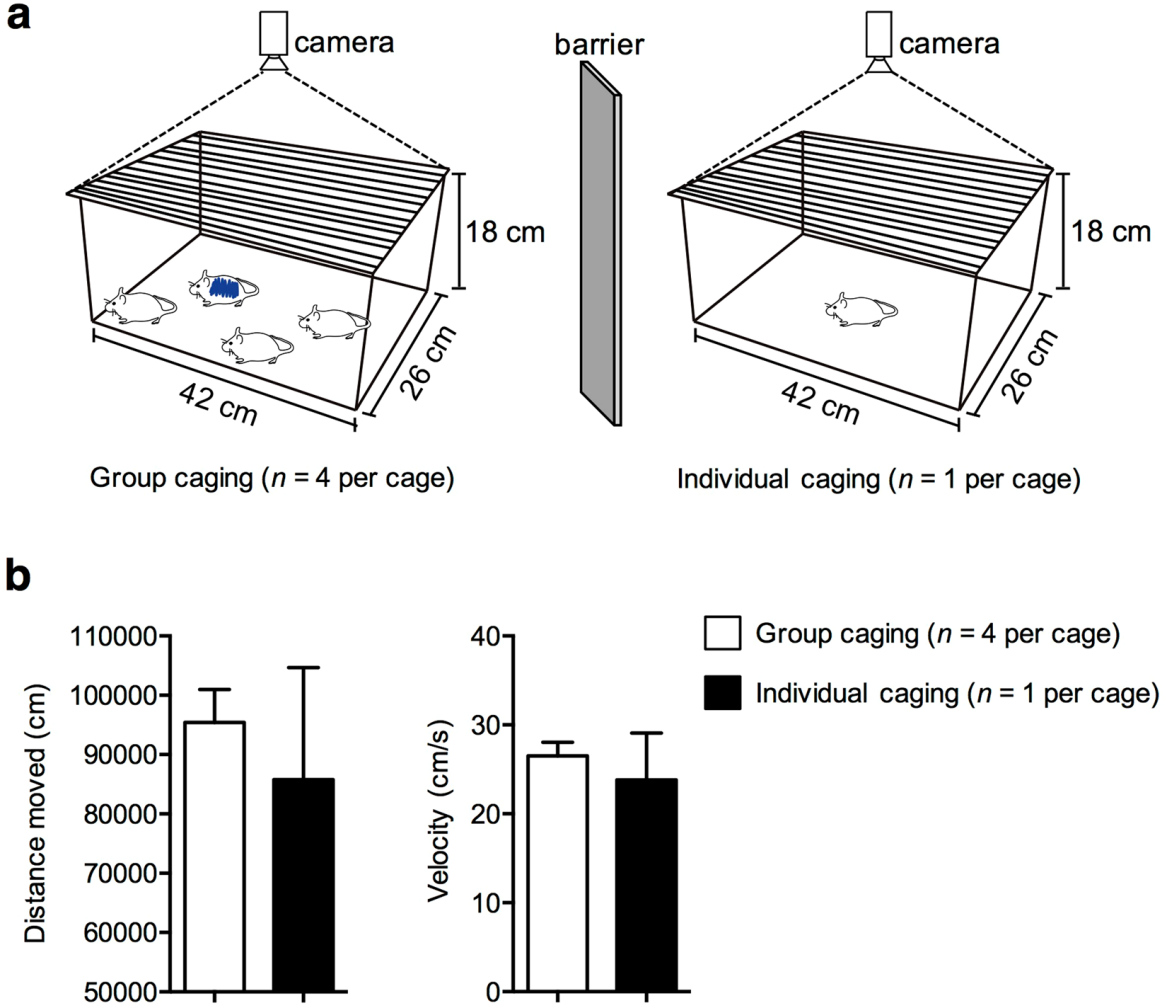
Locomotor activity differences between group-caged and individually-caged mice. Eight-week-old male ICR mice (*n* = 20) were caged in groups of four (*n* = 16) or in individually (*n* = 4). After four weeks, their distance traveled and velocity were measured by the tracking program, EthoVision 3.1. **(a)** The barriers were placed in between the individual and grouped cages and activities of mice were monitored for 15 hours by an overhead tracking camera. An animal marker (blue) was used to stain the back of one randomly selected mouse in each group cage. **(b)** There are no differences in distance moved and velocity in both groups of mice. All the error bars represent the SEM.

We believed that a lack of social interaction could reduce movement of mice. Thus, we predicted that reduced movement may have a role in the changes in BMD, lean tissue percentage, and fat tissue percentage of individually-caged mice. To test our prediction, we monitored the activities of mice in both group and individual cages after four weeks of caging by open field test (Fig. 4a). For group cages, we randomly selected one mouse per cage and stained their backs with an animal marker for tracking. Distance traveled and velocity of each mouse were collected by a tracking program for 15 hours. Against our expectations, there was no significant reduction in movement for individually-caged mice compared to the group-caged mice (Fig. 4b).

## Discussion

In this study, we report a significant increase in fat tissue percentage and a decrease in BMD and lean tissue percentage in individually-caged mice compared to group-caged mice (Fig. 2). This suggests that using different caging conditions – group and individual – may influence the outcome of animal studies, and therefore caging of mice should be made under the same conditions – group or individual. In addition, we observed these physiological changes after a short span of four weeks. This suggests that, if animal studies were conducted over a longer period of time, the different caging conditions will show more noticeable differences in body compositions.

We also report that these alterations in body composition of individually caged mice are not caused by reduced activities (Fig. 4b). Recent studies show that bone degradation occurs in the absence of stimulus, suggesting that the lack of social interactions in individually-caged mice may also contribute to BMD reduction^6,7^. Another study suggests that individual caging of mice may affect the circadian rhythm, including changes in hormone levels in response to stress. These alterations in the central nervous system and immune system may also cause changes in body composition of mice^8^. Thus, further investigation is warranted to stimulus, stress, and other factors that may influence physiological changes in mice belonging to different caging conditions.

Consistent with our outbred ICR mice results, a significant reduction in BMD and lean tissue percentage were previously reported in individually caged young inbred C57BL/6 J mice^9^. This confirms that individual caging of mice can affect body composition despite having different mouse strains. However, our results do not show a change in body mass (Fig. 2a) nor a higher variance in fat tissue percentage and BMD for group-caged mice (Supplementary table S3). We also observed significantly increased fat tissue percentage in individually caged mice.

The ADME process, in particular the metabolism of drugs, is linked to tissue distribution; an increase in fat tissue percentage would interfere with the metabolism of drugs. The alteration of metabolism could hinder drug clearance and absorption^10,11^, which may cause variation and affect the replicability of pharmacological studies. This suggests that if mice, with similar weights but different body compositions, were administered with equal dosages, the effects on each mouse would vary from one another.

The current study shows significant differences in body composition between group- and individual-caged mice. To maintain consistency in animal studies and results, all mice should be placed under identical caging conditions. From the basis of the current study, further investigation is warranted to determine the primary cause of physiological alterations and to observe the long-term effects of differing caging conditions.

## Materials and Methods

### Materials

2,2,2-Tribromoethanol (avertin) and 2-Methyl-2-butanol were obtained from Sigma-Aldrich (Missouri, United States). Saline was purchased from JW Pharmaceutical (Seoul, Korea). The animal marker was purchased from Muromachi Kikai Co., Ltd (Tokyo, Japan).

### Animals

For DXA analysis, eight-week-old male Imprinting Control Region (ICR) mice (*n* = 22) were purchased from Orient Bio Inc. (Seoul, Korea). Twelve mice were caged together in groups of four and the remaining was caged individually for this study. Three cages were prepared for group caging (*n* = 4 per cage) and ten cages were prepared for individual caging (*n* = 1 per cage). For open field test, eight-week-old male Imprinting Control Region (ICR) mice (*n* = 20) were purchased from Orient Bio Inc. (Seoul, Korea) and caged under the same conditions as before. Sixteen mice were caged together in groups of four and the remaining was caged individually. All mice were caged in the same size cages (260 (W) × 420 (D) × 180 (H) mm) for four weeks under regulated conditions with 12-hour light-dark cycles and food and water were provided *ad libitum*. The animal experiments were approved by the Committee for the Care and Use of Laboratory Animals at Yonsei University and performed in accordance with the National Institutes of Health guide for the care and use of laboratory animals.

### Body composition measurement and analysis

The body composition of the mice was measured after four weeks of caging. To prevent subjects from moving during measurement, mice were anesthetized with 4% avertin (v/v) prior to measurement. Body composition parameters of body mass, BMC, BMD, lean and fat tissue of mice were measured using the InAlyzer (Medikors Inc., Korea), which uses dual X-ray absorptiometry (DXA) to provide accurate measurements of bone and body composition for animals. After anesthetization, mice were immediately placed in the center of the InAlyzer scanning area in the prone position with arms and legs extended out to the side. Imaging scans were taken and measurements were obtained using the InAlyzer software.

### Open field test

All mice (*n* = 20) were caged in groups or individually for four weeks and transported to the testing room to acclimate for an hour. Brightness was maintained at approximately 20 lux inside the testing room and barriers were placed in between the individual and grouped cages to prevent interaction. All mice were allowed to roam for 15 hours, and their activities were monitored by an overhead tracking camera, and velocity and distance were tracked using EthoVision 3.1 (Noldus, Netherlands). An animal marker (FG2200B, blue) was used to stain the back of one mouse in the group cages to allow the tracking program to distinguish the marked mouse from the others.

### Statistical analysis

Graphs were obtained with GraphPad Prism 6 and statistical analysis were performed with Student’s *t*-tests (*P < 0.05, **P < 0.01, ***P < 0.001). The error bars represent the SEM.

## Electronic supplementary material

Supplementary Information
