# Supplementary material for: Four-week individual caging of male ICR mice alters body composition without change in body mass
Source: Sci Rep. 2018 Jan 22;8:1331. doi: 10.1038/s41598-018-19808-x (PMC5778080; doi:10.1038/s41598-018-19808-x)

Supplementary Information

Four-week individual caging of male ICR mice alters body composition without change in body mass.

Jisu Shin[1,#], Jiwan Woo[2,#], Yakdol Cho[2,#], Yoon Hee Choi[1], Naewoo Neo Shin[3] and YoungSoo Kim[1,3,*]

[1]Department of Pharmacy, Yonsei University, 85 Songdogwahak-ro, Yeonsu-gu, Incheon 21983, South Korea.

[2]Research Animal Resource Center, Korea Institute of Science and Technology (KIST), Hwarang-ro 14-gil 5, Seongbuk-gu, Seoul 136-791, South Korea.

[3]Integrated Science and Engineering Division, Yonsei University, 85 Songdogwahak-ro, Yeonsu-gu, Veritas Hall D411, Incheon 21983, South Korea

[#]These authors contributed equally to this work.

[*]Correspondence and requests for materials should be addressed to Y.K. (y.kim@yonsei.ac.kr +82-32-749-4523)

Supplementary table S1. Mass, BMC, BMD, lean and fat tissue measurements of group caged mice.

S1

Mouse number	Mass (g)	BMC (g)	BMD (g/cm^2)	Lean (g)	Fat (g)
**1**	46.2	1.23	0.107	37.1	7.91
**2**	47.2	1.27	0.103	40.3	5.65
**3**	47.9	1.18	0.106	36.9	9.78
**4**	47.5	1.30	0.107	37.4	8.83
**5**	42.3	1.20	0.108	36.6	4.42
**6**	45.6	1.22	0.108	37.2	7.12
**7**	45.8	1.19	0.106	36.5	8.08
**8**	40.9	1.12	0.099	33.9	5.89
**9**	50.7	1.46	0.110	41.2	8.05
**10**	46.8	1.32	0.106	38.6	6.93
**11**	46.7	1.23	0.104	38.4	7.10
**12**	47.7	1.38	0.107	39.1	7.21

Supplementary table S2. Mass, BMC, BMD, lean and fat tissue measurements of individually caged mice.

S2

Mouse number	Mass (g)	BMC (g)	BMD (g/cm^2)	Lean (g)	Fat (g)
**1**	48.0	1.24	0.106	35.0	11.8
**2**	45.3	1.15	0.107	32.6	11.6
**3**	53.0	1.38	0.109	40.7	10.9
**4**	48.3	1.26	0.100	37.5	9.6
**5**	43.6	1.15	0.100	33.8	8.6
**6**	48.5	1.26	0.105	36.7	10.6
**7**	47.0	1.20	0.099	39.0	6.8
**8**	44.2	1.16	0.101	35.3	7.7
**9**	46.5	1.18	0.095	38.0	7.4
**10**	48.3	1.20	0.097	38.9	8.2

Supplementary table S3. Mean ± SEM of mass, BMC, BMD, lean and fat tissue in group caged and individually caged mice.

S3

	Mass (g)	BMC (g)	BMD (g/cm^2)	Lean (g)	Fat (g)
**Group caging**	46.3 ± 0.741	1.26 ± 0.027	0.106 ± 0.001	37.8 ± 0.550	7.25 ± 0.420
**Individual caging**	47.3 ± 0.848	1.22 ± 0.022	0.102 ± 0.002	36.7 ± 0.806	9.31 ± 0.577

Supplementary figure S1. Bone, X-ray and body composition images of group caged mice. DXA analysis images of mice.

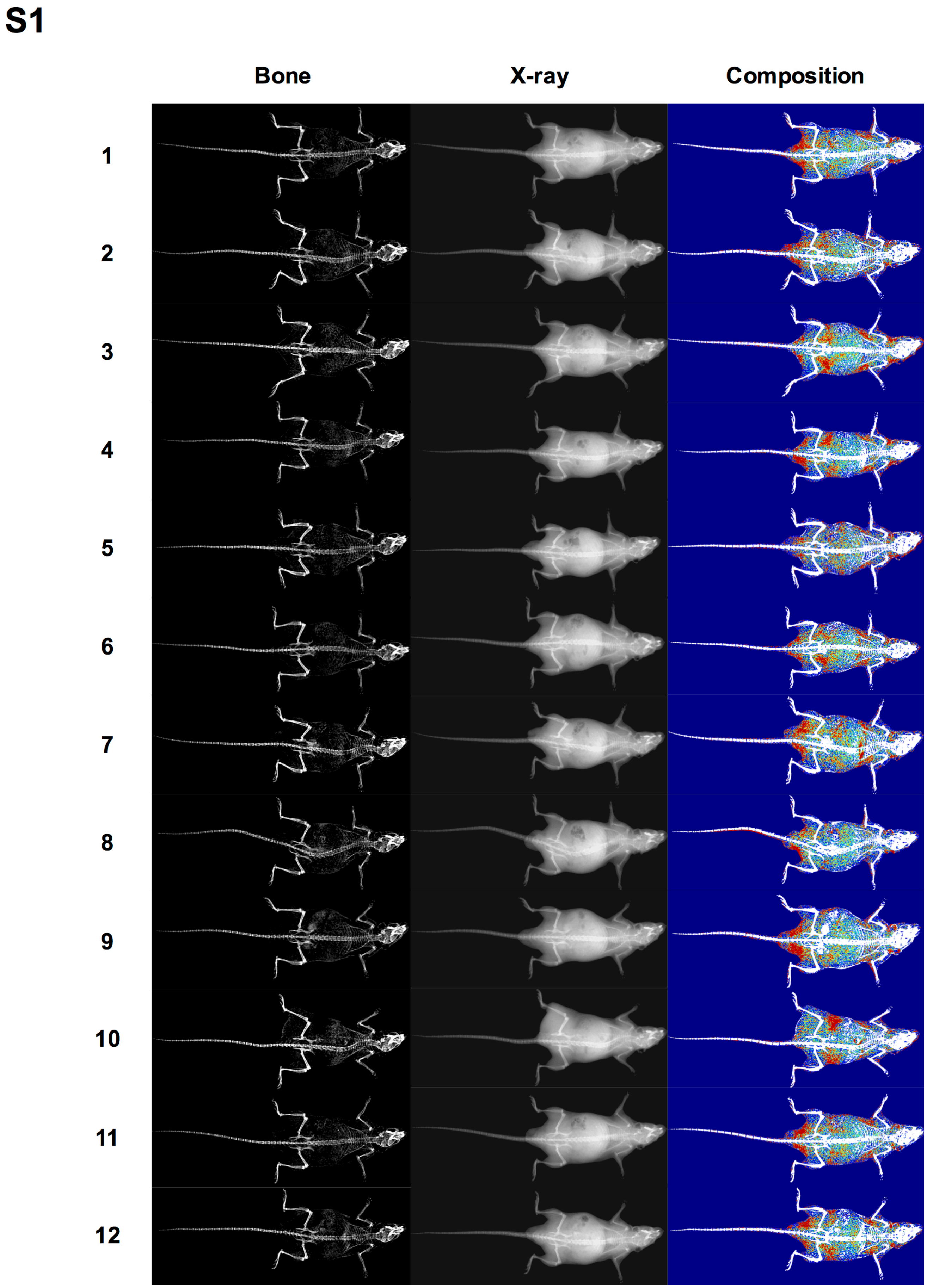

Supplementary figure S2. Bone, X-ray and body composition images of individually caged mice. DXA analysis images of mice.

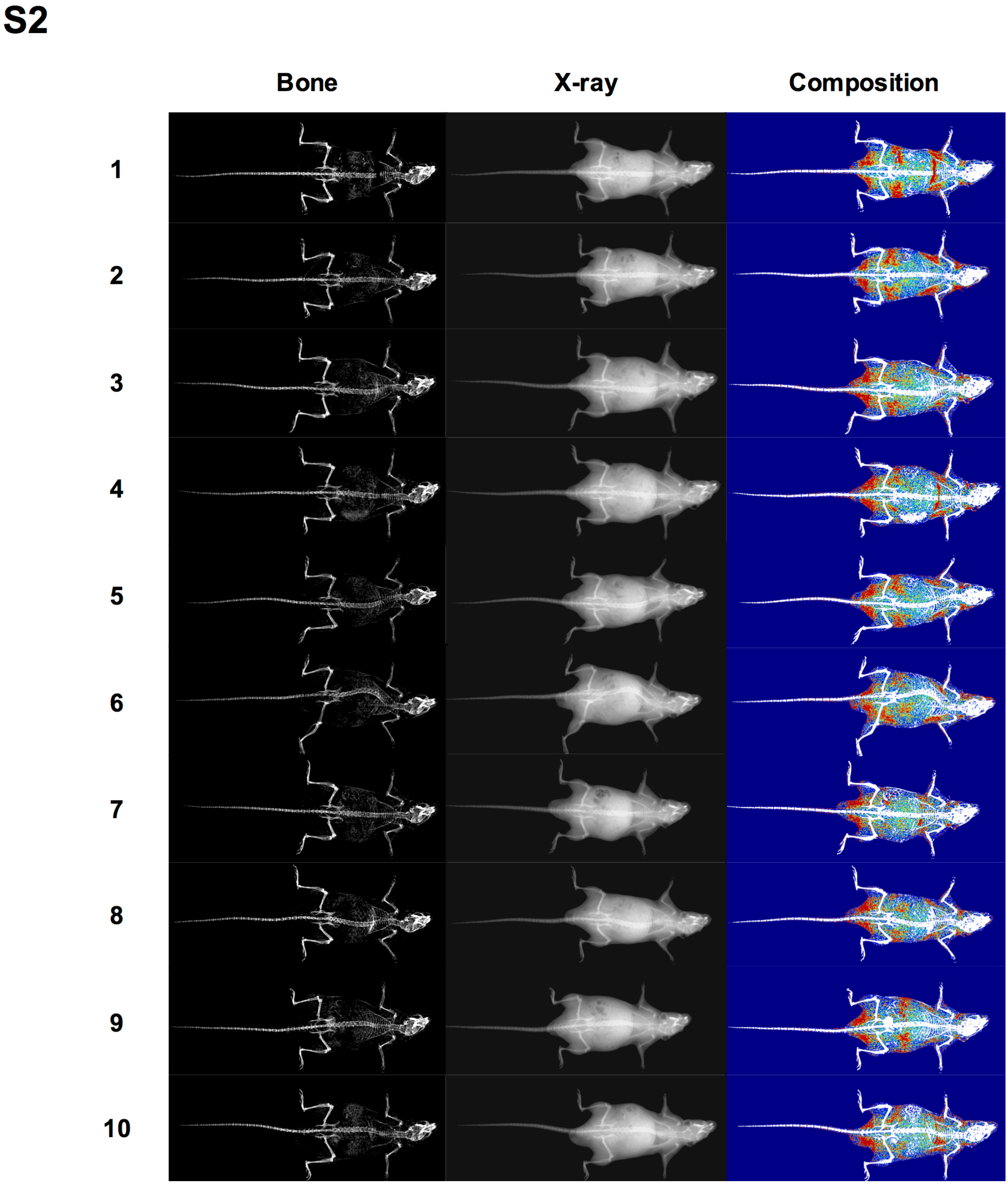

Supplement: Supplementary file 1 — Supplementary Information [file 41598_2018_19808_MOESM1_ESM.pdf]
